# Species composition, environmental determinants, and spatial risk of mosquito breeding sites in urban Libreville, Gabon

**DOI:** 10.1186/s13071-026-07268-6

**Published:** 2026-05-22

**Authors:** Natacha Poungou, Neil Michel Longo Pendy, Boris Makanga, Silas Lendzele Sevidzem, Larson Boundenga, Jacques-François Mavoungou, Rodrigue Mintsa Nguema

**Affiliations:** 1 Laboratoire d’Écologie des Maladies Transmissibles (LEMAT), Université Libreville Nord, BP 1177 Libreville, Gabon; 2 Health Ecology Research Unit (URES), Interdisciplinary Centre for Medical Research of Franceville (CIRMF), BP 769, Franceville, Gabon; 3 Institut de Recherche en Écologie Tropicale (IRET/CENAREST) BP: 13354, Libreville, Gabon; 4 Ecole Doctorale Régionale d`Afrique Centrale, en Infectiologie Tropicale, BP876 Franceville, Gabon

**Keywords:** Mosquitoes, Seasonality, Infection risk, Breeding sites, Libreville airport area

## Abstract

**Background:**

Rapid urbanization in Central African cities creates new habitats for larval mosquitoes and increases the risk of vector-borne diseases. In Libreville, Gabon, the ecological determinants and spatial distribution of these habitats are not well understood. This study assessed species diversity, environmental drivers, emergence success, and spatial risk patterns to inform vector control strategies.

**Methods:**

Larval habitats were surveyed in three urban areas of Libreville during the dry and rainy seasons. Larvae were selected and placed in rearing tanks and emerged adults were identified morphologically. Larval habitat samples were analyzed for conductivity, total dissolved solids (TDS), temperature, redox potential, turbidity, and proximity to dwellings. Redundancy analysis (RDA) was used to explore the associations between environmental variables and species composition. Logistic regression identified the determinants of larval emergence success. Spatial risk was mapped using inverse distance weighting (IDW).

**Results:**

A total of 1909 larvae from 18 species across 5 genera were recorded. Larval abundance differed significantly between seasons with higher count recorded during the dry season (Wilcoxon rank-sum test: *W* = 3333.5, *P* = 0.0169). RDA explained 23.5% of the variation in species, with conductivity and total dissolved solids (TDS) identified as key drivers. *Aedes* species were found to prefer low-mineralized waters, whereas *Culex* and *Anopheles* species were found to be associated with ion-rich habitats. Emergence success increased with water temperature (around 33 °C) and redox potential. However, the rainy season was associated with a slight decrease in emergence probability compared with dry season, although it was not statistically significant (*P* = 0.084). Spatial analysis revealed heterogeneous risk, with hotspots near the airport, Okala, and Alibandeng. No major differences were found between artificial and natural habitats.

**Conclusions:**

Urban larval mosquito communities in Libreville exhibit strong seasonal and environmental structuring, with defined breeding hotspots. Conductivity and total dissolved solids (TDS) predict assemblage composition, while temperature and habitat stability affect emergence. Integrating ecological and spatial analyses provides valuable insight for targeted vector control in Central African cities.

**Supplementary Information:**

The online version contains supplementary material available at 10.1186/s13071-026-07268-6.

## Background

Vector-borne diseases remain a leading public health challenge worldwide, particularly in tropical and subtropical regions, where the climate and ecology conditions favor the proliferation of vectors [1–3]. Mosquitoes are of particular concern because they transmit the pathogens responsible for malaria, arboviruses (e.g., dengue, chikungunya, Zika, and yellow fever), and filariasis [3–5]. These diseases impose a substantial health and socioeconomic burden, especially in Africa, where health systems are already under pressure from multiple endemic and emerging diseases [6]. Rapid and often unplanned urbanization across the African continent has profoundly transformed natural ecosystems, creating novel and heterogeneous breeding habitats for mosquito vectors [7–9]. Artificial containers such as discarded tires, household water storage tanks, and drainage systems provide stable larval habitats in close proximity to human populations [3, 10]. This shift in habitat availability not only sustains dense and diverse mosquito populations, but also promotes the coexistence of multiple vector species within confined urban environments, amplifying the risk of co-circulating pathogens and coinfections [11].

Central Africa is currently undergoing major demographic and environmental transition, with cities such as Libreville (Gabon) experiencing rapid population growth, land-use changes, and increasing ecological fragmentation. These transformations increase the abundance of mosquito breeding sites and alter their physicochemical properties, which directly influence larval development, adult emergence, and vector population dynamics [3, 12]. Although several studies have investigated the larval ecology of *Anopheles* in urban environments [10, 12], much less attention has been given to the broader assemblages of *Aedes* and *Culex*, despite their major role in arbovirus and filarial transmission. Furthermore, spatial heterogeneity in larval habitats remains poorly documented in Central African cities, despite its importance for predicting hotspots of entomological risk and informing integrated vector management (IVM) strategies [13–15].

Due to its unique ecological setting on the Atlantic coast, its rapid urban sprawl, and its high receptivity to vector-borne diseases [12], Libreville represents a critical case study for understanding the eco-epidemiology of mosquito populations at the human–environment interface. Identifying the ecological drivers of larval habitat suitability and mapping their spatial risk distribution are essential to guiding targeted and sustainable control interventions [16–18]. This study therefore integrates ecological, physicochemical, and spatial analyses to (i) document the diversity and seasonal dynamics of mosquito communities in urban Libreville, (ii) identify the environmental determinants of larval community structure and emergence success, and (iii) map the spatial distribution of entomological risk across neighborhoods. By combining entomological ecology with geospatial risk modeling, our approach provides actionable insights that can inform context-specific control strategies, aligning with both One Health frameworks and the global agenda for sustainable urban health.

## Methods

### Study area

**Fig. 1:**
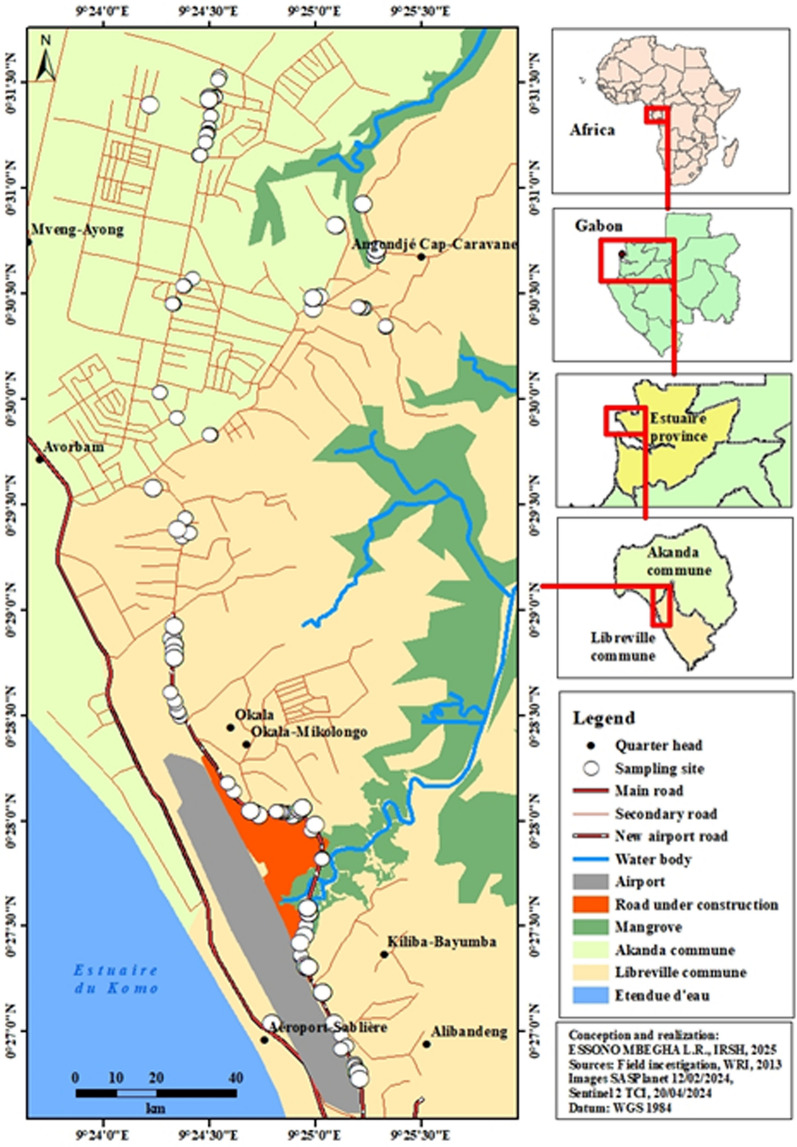
Geolocalization of sampled mosquito habitats

The study was conducted in Libreville (Gabon), in the urban zone located behind the Léon-Mba International Airport. This area has undergone significant topographical modifications due to the construction of a bypass road, which has altered water drainage patterns and contributed to the creation of new larval habitats near human dwellings. Sampling took place during both the dry and rainy seasons. During the construction of this airport road in the dry season, the road construction companies frequently watered the area. This practice serves two purposes: first, to minimize dust that could affect nearby populations and their own employees; and second, to facilitate soil compaction by heavy machinery. As a result, these activities probably multiplied puddles along the road segment, creating ideal colonization sites for *Anopheles* larvae from eggs laid by adult females in these newly created habitats. All aquatic habitats with potential to harbor mosquito larvae were systematically surveyed and georeferenced using Global Positioning System (GPS) during two standardized sampling campaigns conducted in the dry season (February 2024) and the rainy season (November 2024), each lasting 12 consecutive days (Fig. 1). Larval habitats were classified on the basis of their nature (e.g., puddles, canals, artificial containers, drainage structures, etc.).

### Larval habitat sampling and environmental measurements

Mosquito larvae were sampled using the standard dipping method widely employed in vector ecology, with five dips per breeding site performed using a 350 ml standard dipper [19, 20]. Larvae were transferred to labeled containers and brought to the laboratory for rearing until adult emergence. For each breeding site, the following physicochemical parameters were measured in situ and in the laboratory during rearing days: (i) quantitative variables: water temperature (°C), pH, electrical conductivity (µS), total dissolved solids (TDS, ppm), salinity (ppm), turbidity (NTU), oxidation–reduction potential (ORP, mV), dissolved oxygen (mg/L), and water depth (cm) were measured using the microprocessor water and soil analysis kit (8 parameters) model no. S-959 (SYSTONIC(s) Lab and Scientific Instruments India); (ii) qualitative variables: presence or absence of aquatic vegetation, green algae, and organic matter; water type (stagnant or flowing); and water clarity (clear, slightly turbid, or turbid). The distance between each larval site and the nearest inhabited building was measured on site using a measuring tape. In the laboratory, larvae were reared under standardized conditions until adult emergence. Emerged mosquitoes were then morphologically identified as genus, and when possible, to species level using standard entomological keys [21–24]

### Biostatistical analyses

All statistical analyses were conducted using R version 4.4.0. A logistic regression model (glm, binomial family) was applied to evaluate the effects of environmental variables on the binary outcome of emergence success. Model selection was based on the Akaike information criterion (AIC) using stepwise backward elimination via the *step* function from the *MASS* package [25]. The significance of predictors such as temperature, redox potential (ORP), and season was tested, and marginal effect plots were generated with the effects package and visualized using *ggplot2*. To explore the relationships between environmental gradients and mosquito species composition, redundancy analysis (RDA) was performed using the *vegan* package [26]. Abundance data were Hellinger-transformed using *decostand*. Only continuous environmental variables were included to meet the assumptions of *RDA*. The significance of environmental constraints and RDA axes was tested by 999 permutations. The ordination plot was constructed with *ggplot2* and enhanced using *ggrepel*.

The entomological risk associated with larval habitats was assessed in three stages: (i) comparative analysis of observed risk (based on larval density and emergence success) across breeding sites, with Wilcoxon test used to assess statistical differences; (ii) mapping of observed vector risk, incorporating urban road networks (*osmdata* [27]), breeding site locations (sf), and highlighting the airport zone (red polygon) [28]; we developed a composite vector risk index to estimate the potential transmission risk associated with each larval habitat, which integrated three measured parameters: larval density, female-to-male ratio, and distance to the nearest dwelling, and was defined as follows: vector risk index = larval density × female ratio (1/distance to dwelling + 1); and (iii) prediction of spatial risk was performed using inverse distance weighting (IDW) interpolation via the *gstat* package. Raster outputs were visualized using ggplot2, applying perceptually uniform color palettes (viridis, option “plasma”).

## Results

### Mosquito species diversity and seasonal dynamics

Larval sampling led to the collection of 84 samples in the dry season and 64 in the rainy season. A total of 1909 larvae were obtained from these samples, representing 18 species from 5 genera (*Aedes*, *Anopheles*, *Culex*, *Lutzia*, and *Toxorhynchites*) were identified across urban habitats in Libreville (Table 1). *Culex* genus was the dominant genus (46.7%, n/N), followed by *Aedes* (31.1%, n/N) and *Anopheles* (22.0%, n/N), while *Lutzia* and *Toxorhynchites* were rare. Species distribution differed by season: larval abundance differed significantly between seasons with higher count recorded during the dry season (Wilcoxon rank-sum test: *W* = 3333.5, *P* = 0.0169). However, this difference was small in magnitude (Cliff’s *δ* = 0.23; 95% CI 0.04–0.40). The occurrence of species exhibited marked temporal heterogeneity. Sampling conducted in February (mid-dry season) and November (peak rainy season) 2024 revealed that some species were detected exclusively during one sampling period. For example, *Aedes aegypti* and *Culex simpsoni* were only recorded in the November survey, whereas *Culex trifilatus* and *Lutzia tigripes* were only observed in February. Other species, including *Aedes albopictus* and *Anopheles gambiae* s.l., were detected during both surveys, but exhibited higher abundance in February (Table 1).

**Table 1:** Variation in the number of different species of mosquito according to the season

Mosquito species	Dry season	Rainy season	Total
*Aedes*	327	267	594
*Aedes aegypti*	0	135	
*Aedes albopictus*	327	132	
*Anopheles*	282	138	420
*Anopheles gambiae s*.l	282	138	
*Culex*	765	126	891
*Culex (Culiciomyia) sp*	53	8	
*Culex cinerellus*	54	3	
*Culex cinereus*	1	13	
*Culex decens*	84	7	
*Culex duttoni*	225	21	
*Culex gaillardi*	1	0	
*Culex macfei*	1	0	
*Culex perfuscus*	0	11	
*Culex quinquefasciatus*	124	11	
*Culex simpsoni*	0	14	
*Culex sp*	193	29	
*Culex thalassius*	0	9	
*Culex trifilatus*	29	0	
*Lutzia*	3	0	3
*Lutzia tigripes*	3	0	
*Toxorhynchites*	0	1	1
*Toxorhynchites sp*	0	1	
Total	1377	532	1909

### Microenvironmental drivers of community composition

**Fig. 2:**
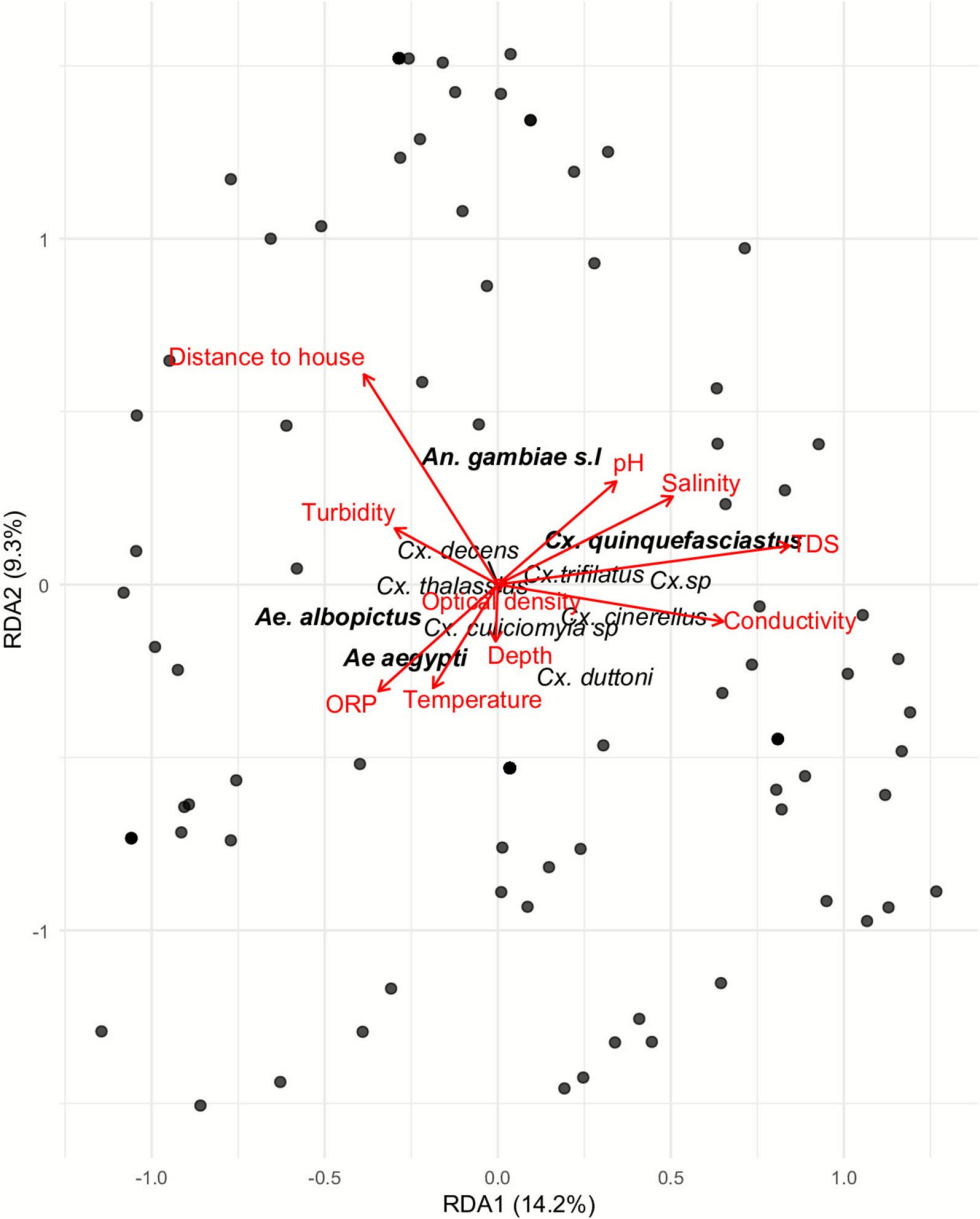
Mosquito community structure as a function of physicochemical parameters

Redundancy analysis (RDA) was used to assess the influence of the physicochemical parameters of the breeding sites on the species composition of mosquitoes emerging in an urban environment (Fig. 2). The model explained approximately 23.5% of the total variance in the data, of which 14.2% was explained by the first canonical axis (RDA1) and 9.3% by the second (RDA2). Among the environmental variables tested, conductivity (us) emerged as the only statistically supported discriminating factor (*F* = 2.21; *P* = 0.050). Total dissolved solids (TDS; ppm) showed a weaker contribution that was not statistically significant (*p* = 0.09), indicating a secondary trend that was consistent with the conductivity gradient, but which did not reach conventional levels of statistical significance. The species *Aedes albopictus* (AAL) and *Aedes aegypti* (AAE) were located on the negative side of RDA1, indicating a preference for low mineral deposits. Conversely, the genera *Culex* and *Anopheles* appeared to be more associated with waters richer in dissolved ions. Redox potential, turbidity, and distance to dwellings displayed secondary associations with community structure, demonstrating marginal or insignificant effects in comparison with conductivity (*p* > 0.05) (Fig. 2). The overall ordering thus highlights a partial ecological segregation of species according to local environmental gradients, reflecting a differentiation in their larval preferences.

### Determinants of larval development success

**Fig. 3:**
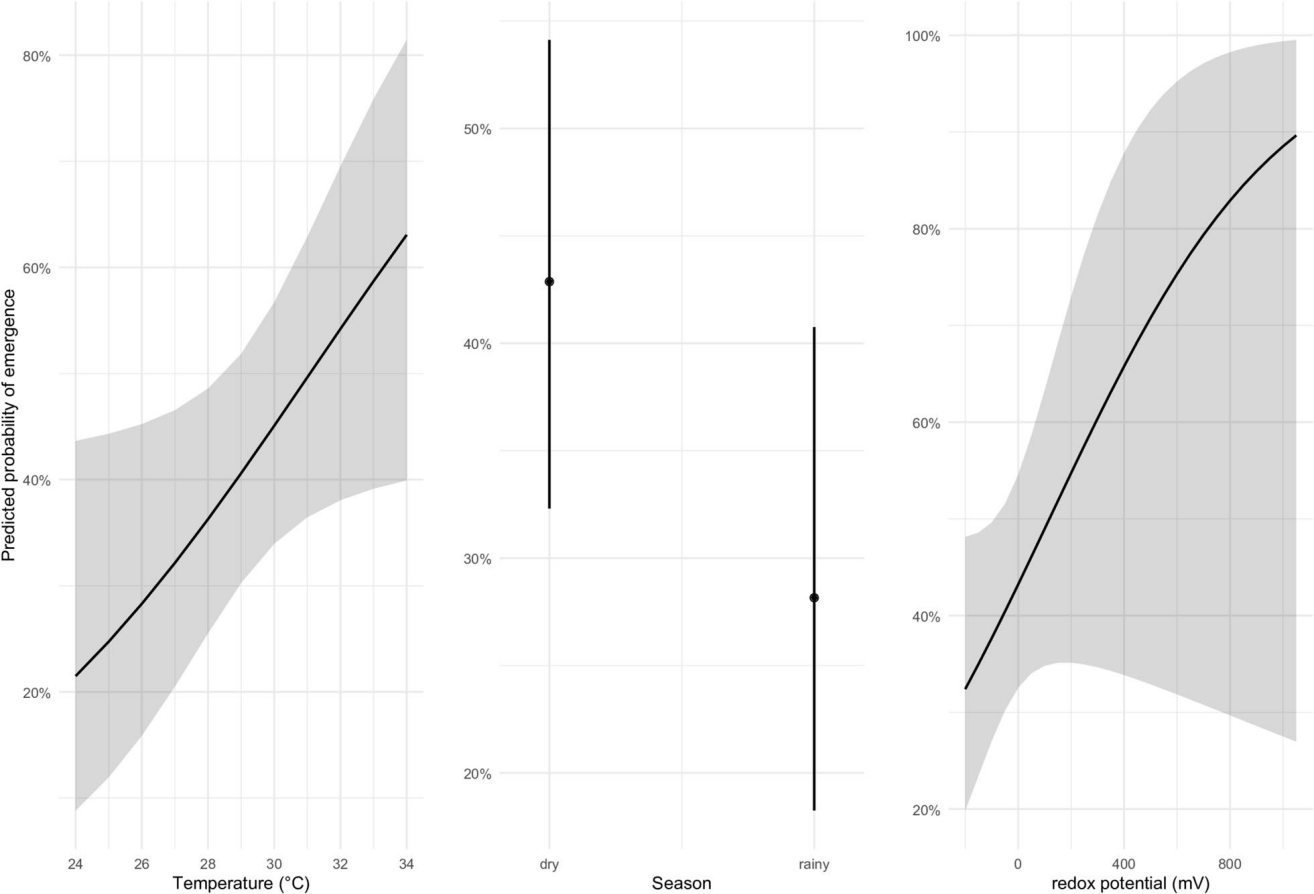
Factors influencing mosquito emergence success: results from the logistic regression model

The final logistic regression model (AIC = 187.66) identified water temperature, season, and redox potential (ORP) as key predictors of larval development success (Table 2). Water temperature was found to have a significant positive effect on emergence probability (*β* = 0.183, OR 1.20, 95% CI 1.01–1.44, *P* = 0.042). The effect of water temperature on emergence probability peaked at 34 °C, with predicted emergence success exceeding 60% (Fig. 3). Emergence success tended to be lower during the rainy season than during the dry season, though this difference was not statistically significant (*β* = −0.65, OR 0.52, 95% CI 0.25–1.10, *P* = 0.084). Redox potential showed a weak, nonsignificant positive association with emergence success (*β* = 0.002, OR approximately 1, *P* = 0.116) (Table 2). These results suggest that both abiotic stressors and seasonal fluctuations modulate mosquito survival through to adulthood, with temperature exerting the strongest effect (Fig. 3).

**Table 2:** Logistic regression analysis of factors associated with mosquito larval emergence

Predictor	Estimate (*β*)	SE	*z*-Value	Odds ratio (OR)	95% CI (OR)	*P*-value
Intercept	−5.674	2.650	−2.141	0.003	0.000–0.38	0.032
Season (rainy versus dry)	−0.650	0.376	−1.726	0.52	0.25–1.10	0.084
Water temperature (°C)	0.183	0.090	2.032	1.20	1.01–1.44	0.042
Redox potential (ORP)	0.002	0.001	1.571	1.00	1.00–1.01	0.116

### Spatial heterogeneity of entomological risk

**Fig. 4:**
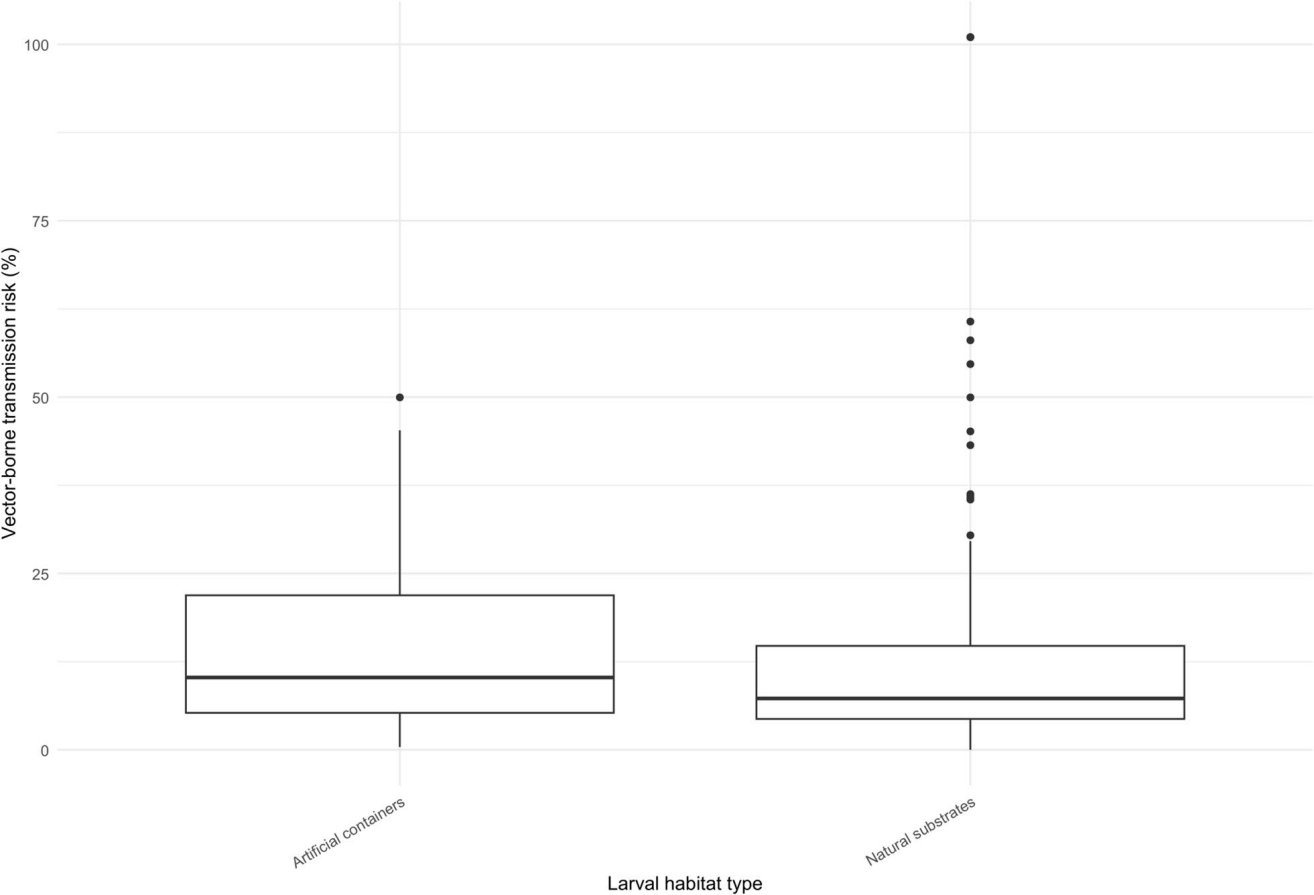
Mosquito-borne transmission risk based on breeding site-types

**Fig. 5:**
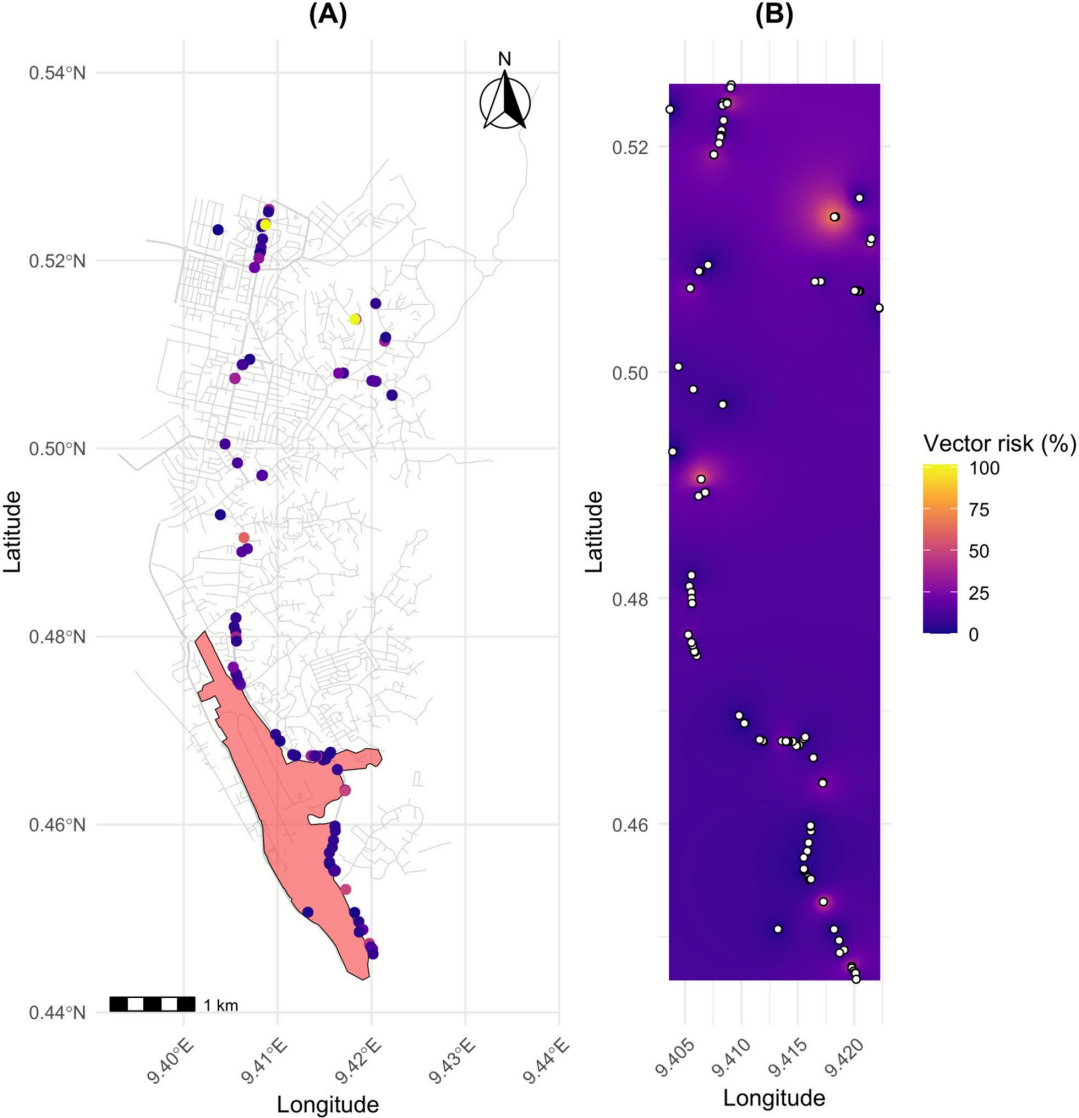
Spatial distribution of mosquito-borne transmission risk

Entomological risk varied significantly across the three surveyed districts. Although artificial habitats exhibited slightly higher risk values than natural ones, no statistical difference was detected (*P* > 0.05; Wilcoxon test; Fig. 4). Spatial mapping revealed that the highest risk clusters were located near Léon-Mba International Airport, as well as in Okala and Alibandeng (Fig. 5A). Inverse distance weighting (IDW) interpolation further highlighted persistent hotspots across the entire study area (Fig. 5B). This spatial heterogeneity underscores that mosquito proliferation is not evenly distributed but concentrated in specific urban microenvironments, often coinciding with human activity hubs and transport infrastructures.

## Discussion

In rapidly urbanizing Central African cities, mosquitoes that transmit diseases represent a persistent and growing health risk. Our study shows that the distribution of mosquitoes in Libreville is not random, but is strongly influenced by seasonal dynamics, physicochemical gradients, and spatial heterogeneity. This emphasizes the urgent need for locally tailored control strategies that move beyond generic, insecticide-based methods [29].

### Species diversity and seasonal variations

We identified 18 mosquito species belonging to 5 genera (*Aedes*, *Anopheles*, *Culex*, *Lutzia*, and *Toxorhynchites*), which highlights the remarkable ecological plasticity of mosquitoes in human-modified urban environments. The dominance of *Culex* and *Aedes* (46.7% and 31.1% of the individuals collected, respectively) likely reflects their ability to exploit a variety of artificial larval habitats, such as containers and organically enriched waters, which are prevalent in rapidly urbanizing areas [30, 31]. There were marked seasonal contrasts in species occurrence with some taxa being detected exclusively during either the dry or rainy seasons. Rather than reflecting fixed seasonal preferences, these patterns likely result from dynamic interactions between rainfall intensity, habitat stability, and short-term hydrological processes, such as flushing and desiccation, which can rapidly modify larval habitat suitability [32, 33]. In equatorial urban environments, where seasonal boundaries are often indistinct, mosquito populations are therefore expected to exhibit significant variability within the season that may not be fully captured by binary seasonal classifications. These observations highlight the limitations of single-season larval surveys and presence/absence-based assessments. Turnover of species across and within seasons can substantially alter local transmission potential, particularly when shifts involve vectors that are important from an epidemiological perspective, such as *Anopheles gambiae* s.l. or *Aedes* spp. The contribution of these species to disease risk is not equivalent to that of non-vector or predatory taxa, such as *Toxorhynchites* spp. Consequently, vector surveillance and risk assessment strategies should incorporate temporally resolved sampling frameworks that account for both species composition and vectorial relevance [34, 35].

### Physicochemical determinants of community’s structure

Redundancy analysis (RDA) revealed that the conductivity and total dissolved solids (TDS) were the strongest predictors of community composition, accounting for much of the ecological segregation observed. *Aedes aegypti* and *Ae. albopictus* preferentially colonized low-mineralized waters, consistent with their global adaptation to rain-fed containers [36–40], while *Culex* and *Anopheles* were more associated with ion-rich, stagnant, and anthropogenic waters [8, 41]. These findings are consistent with previous reports urban pollutants and ionic enrichment create selective niches that sustain highly competent vectors such as *Cx. quinquefasciatus* [8, 12, 41]. However, our study also highlights that species-specific responses are not fixed: local selective pressures, including salinity tolerance in coastal environments, may shift larval preferences, complicating predictive models.

### Abiotic and seasonal drivers of emergence success

The logistic regression model identified water temperature, season, and redox potential as significant predictors of larval development success. Emergence probability peaked at 34 °C, aligning with known thermal optima for accelerated development of *Aedes* and *Culex* larvae [42–44]. The effect of season on emergence success did not reach conventional statistical significance (*P* = 0.084); the direction of the association suggests a context-dependent seasonal modulation rather than a strong deterministic effect. The lower emergence probability observed during the rainy season is biologically plausible and likely reflects short-term hydrological disturbances, including flushing of breeding sites, dilution of nutrient resources, and increased predator pressure, which can transiently reduce larval survival [45, 46]. Conversely, dry-season stability promotes organic matter accumulation and predator scarcity, favoring larval development. These findings underscore the importance of considering microenvironmental stability, not just mean climatic conditions, in vector ecology.

### Spatial heterogeneity and public health implications

A spatial analysis revealed heterogeneous vector risk, with hotspots concentrated near Léon-Mba International Airport, Alibandeng, and Okala. The clustering of high-risk habitats near the airport is of particular concern, as it may facilitate the dispersal of invasive species and pathogens through air travel [47–49]. The absence of differences between natural and artificial habitats further emphasizes that urban mosquitoes exploit all available niches, demanding integrated interventions that target both domestic containers and periurban wetlands [43, 44]. Spatial risk modeling thus provides actionable tools for municipal authorities, enabling hotspot-targeted larval source management, optimized allocation of limited resources, and early warning systems for vector-borne outbreaks [42].

Despite the insights it provides, this study has several limitations. Sampling was restricted to three urban districts of Libreville, which limits the spatial generalization of the findings. Additionally, larval identification primarily relied on morphological characteristics, which could have resulted in an underestimating of cryptic diversity within taxonomically complex groups, such as the *An. gambiae* complex and *Culex* spp. Therefore, future studies should integrate molecular tools, such as COI and ITS2 barcoding, to improve taxonomic resolution and strengthen ecological interpretations [12, 50]. Furthermore, the role of interspecific interactions, species richness, and larval density-dependent processes in shaping mosquito community structure and emergence success was not explicitly assessed. In aquatic larval habitats, competitive asymmetries and predator–prey interactions can strongly influence population dynamics. For instance, the presence of predatory larvae, such as those of the genus *Toxorhynchites*, can substantially reduce the survival and emergence probability of other mosquito species through intraguild predation. Similarly, differences in competitive ability among co-occurring species may lead to nonlinear effects of larval density on development and survival. The absence of these factors from the analytical framework may therefore mask important biotic mechanisms underlying the observed patterns. Future investigations should incorporate community-level metrics (for example, species richness, diversity indices, and species accumulation curves) and explicitly model biotic interactions to provide a more mechanistic understanding of larval mosquito ecology. Additionally, our entomological risk index did not take into account the infection status of adult mosquitoes. However, the epidemiological relevance of the larval habitats identified in this study can be inferred from the well-documented vectorial roles of the dominant species recorded. *Aedes aegypti* and *Aedes albopictus* are well-known vectors of arboviruses such as dengue, chikungunya, and Zika [51–53], while *Anopheles gambiae* s.l. is the main malaria vector [54–56] in Central Africa. Therefore, the spatial concentration of productive larval habitats harboring these species represents a credible proxy for transmission potential, even in the absence of direct pathogen detection. Nevertheless, future integration of larval habitat mapping with pathogen screening of emerging adults would strengthen the link between entomological indicators and epidemiological risk, enabling a more precise assessment of active transmission foci [40, 50].

## Conclusions

This study provides one of the first integrated ecological and spatial assessments of mosquito breeding sites in an urban Central African context. By combining larval community composition data, physicochemical characterization of breeding sites, and geospatial analyses, we demonstrate that urban mosquito populations in Libreville are structured by interacting environmental gradients, habitat instability, and marked spatial heterogeneity. Our findings highlight three critical messages. First, the composition of larval communities is strongly influenced by physicochemical filtering, particularly conductivity, total dissolved solids (TDS), and water temperature, as *Aedes* species were primarily associated with low-ion, rain-fed habitats, whereas *Culex* and *Anopheles* species were more frequently linked to ion-rich, organically enriched waters. This reflects niche differentiation within urban breeding environments. Second, temporal patterns in larval abundance and emergence success were not uniformly seasonal but instead reflected habitat-specific responses to rainfall dynamics. Intense precipitation likely reduced larval persistence through flushing and habitat disturbance, whereas stable conditions favored development, highlighting the limitations of dry rainy season classifications in urban tropical settings. Third, spatial analyses revealed pronounced heterogeneity in entomological risk, with discrete larval production hotspots concentrated in densely populated neighborhoods and areas of high human mobility, particularly around transport infrastructure. Together, these findings emphasize that the entomological risk in urban environments is neither spatially nor temporally homogeneous, but rather emerges from the interaction between the ecology of different species, environmental conditions, and the structure of the landscape. Integrating ecological monitoring with spatial hotspot mapping can substantially improve the efficiency of larval source management, enabling targeted, context-specific interventions. In rapidly urbanizing African cities such as Libreville, it is essential to incorporate such approaches into existing malaria and arbovirus control programs, particularly in high-risk zones associated with human movement. More broadly, this study highlights the importance of adopting a One Health approach that recognizes the close relationship between urban environmental change, vector ecology, and human health.

## Supplementary Information

Supplementary Material 1.

## Data Availability

Data supporting the main conclusions of this study are included in the manuscript.
