# Supplementary material for: Species composition, environmental determinants, and spatial risk of mosquito breeding sites in urban Libreville, Gabon
Source: Parasit Vectors. 2026 May 22;19:215. doi: 10.1186/s13071-026-07268-6 (PMC13195840; doi:10.1186/s13071-026-07268-6)
Supplement: Supplementary file 1 — Supplementary Material 1. [file 13071_2026_7268_MOESM1_ESM.pdf]

UNIVERSITE LIBREVILLE NORD
UNIVERSITY OF NORTH LIBREVILLE
****
FACULTE DES TECHNOLOGIES ET MANAGEMENT DE LA SANTE
FACULTY OF TECHNOLOGIES AND HEALTH MANAGEMENT
****
Département de Santé Environnement
Department of Environmental Health
BP 1177 Libreville
Tel : +241076302222
Web : www.uiv-ln.academy

REPUBLIQUE GABONAISE
******
UNION TRAVAIL JUSTICE

Libreville, the 17-12-2025

SEVIDZEM Silas Lendzele
Faculty of Technologies and Health Management
University Libreville Nord
Department of Environmental Health
BP 1177 Libreville
Tel: +241 65 23 14 45

To
Editor-in-Chief of ***Parasites & Vectors***

Resubmission of manuscript entitled **« Species Composition, Environmental Determinants and Spatial Risk of Mosquito Breeding Sites in Urban Libreville, Gabon”**

Dear Sir,

I am writing on behalf of authors to resubmit our revised manuscript for publication in your prestigious journal.

We wish to thank the Editor and the two Reviewers for the quality of their review that will go a long way to improve on the quality of our manuscript.

The manuscript has been revised as per the recommendations of the two reviewers and all the modified sections highlighted in yellow ink in the revised version. We have also prepared a step-by-step report on the responses of reviewer comments.

As we are from a low-income country and the first author is a PhD student, we hope our manuscript be revised and published with complete APC waive and rapidly so that the student can defend her thesis on time.

Dr SEVIDZEM Silas Lendzele
